# *Smilax glabra* Roxb. Inhibits Collagen Induced Adhesion and Migration of PC3 and LNCaP Prostate Cancer Cells through the Inhibition of Beta 1 Integrin Expression

**DOI:** 10.3390/molecules25133006

**Published:** 2020-06-30

**Authors:** Oh Yun Kwon, Sujin Ryu, Jong Kyu Choi, Seung Ho Lee

**Affiliations:** Department of Nano-Bioengineering, Incheon National University, 119 Academy-ro, Yeonsu-gu, Incheon 22012, Korea; ohyun1220@naver.com (O.Y.K.); lovedbtnwls@naver.com (S.R.); carmonster@naver.com (J.K.C.)

**Keywords:** *Smilax glabra* Roxb., prostate cancer, integrin beta 1, migration, FAK phosphorylation

## Abstract

*Smilax glabra* Roxb. (SGR) has been used as a traditional medicine for brucellosis and syphilis. In this study, we investigated whether nontoxicological levels of water extract of SGR (WESGR) are effective for suppressing steps in the progression of prostate cancer, such as collagen-mediated migration and adhesion and identified the target molecule responsible for such effects. We found that nontoxicological levels of WESGR did not attenuate PC3 and LNCaP cell adhesion to serum but did significantly do so with collagen. In addition, using the Boyden chamber assay, we found that nontoxicological levels of WESGR did not inhibit the migration of PC3 and LNCaP cells to a serum-coated area but did significantly attenuate migration to a collagen-coated area. Interestingly, the expression of α2β1 integrin, a known receptor of collagen, was not affected by ectopic administration of WESGR. However, WESGR significantly attenuated the expression of β1 integrin, but not α2 integrin when PC3 and LNCaP cells were placed on a collagen-coated plate, resulting in attenuation of focal adherent kinase phosphorylation. Finally, 5-*O*-caffeoylquinic acid was determined as a functional single component which is responsible for antiprostate cancer effects of WESGR. Taken together, our results suggest a novel molecular mechanism for WESGR-mediated antiprostate cancer effects at particular steps such as with migration and adhesion to collagen, and it could provide the possibility of therapeutic use of WESGR against prostate cancer progression.

## 1. Introduction

Prostate cancer is the second most common cancer in males, with reports stating that over 80% of cases are detected after age 65 [1]. If prostate cancer is detected in an early stage, it is considered curable with any of several treatments, including prostatectomy and androgen deprivation therapy (ADT) [2]. However, prostate cancer patients recurrently treated with ADT can develop an incurable disease state called castration-resistant prostate cancer (CRPC). Although majority of metastatic disease patients are responsive to hormone deprivation therapy, CRPC can grow without testosterone, and most cases of CRPC develop into metastatic prostate cancer, which is responsible for high mortality rates in prostate cancer patients [3,4]. Metastatic prostate cancer migrates primarily to the lymph nodes and the skeleton. When cancer cells initiate metastasis, small populations of the primary tumor invade the surrounding tissues and then intravasate the circulatory system. Migrating cancer cells eventually colonize distant organs by interacting with the extracellular matrix (ECM). Since most chemotherapeutic agents for cancer patients have been developed to attenuate cancer cell proliferation and/or induce apoptosis, relatively high doses of agents are often used for cancer treatment, and this fact could induce cytotoxicity in normal cells, resulting in severe side effects. Therefore, the development of anticancer therapeutics with lower cytotoxicity and that target the inhibition of metastatic events such as cell migration and invasion could lead to an effective cure for prostate cancer, particularly metastatic CRPC (mCRPC).

Integrin is a well-known heterodimeric transmembrane receptor responsible for cell-to-ECM interactions. They are composed of α and β subunits that make up 24 unique αβ complexes possessing distinct ligand-binding properties [5,6]. When an integrin interacts with its ligand, such as collagen or fibronectin, it undergoes a conformational change to produce the ligand-binding specificity [7] and activates various intracellular signaling, such as focal adhesion kinase (FAK) molecules. Such signaling is known to contribute to lamellipodium formation, cell adhesion, and cell migration on the ECM [8]. Integrins have been a focus of research as a potential target for the development of anticancer therapeutics because it has been reported that the expression of integrins correlates with stages of human cancers [9,10]. Although there are several kinds of integrin expression in prostate cancer, α2β1, a receptor for collagen I, seems to be most abundantly expressed in prostate cancer cells [11] and appears to have a more important role in the invasion process of PC3 cells than other kinds of integrin [12,13]. In addition, Bonkhoff et al. reported that the expression of α2β1 integrin is upregulated in lymph node metastases as compared to primary prostate tumors [14], suggesting that α2β1 integrin is a potential therapeutic target for prostate tumorigenesis, especially for mCRPC.

*Smilax glabra* Roxb. (SGR) is a traditional folk medicine that has been used in the treatment of hyperglycemia and for detoxication [15,16]. SGR has been reported to have various bioactivities, including antiviral [17], anti-inflammatory [18], and immunomodulatory activities [18,19]. Moreover, an anticancer activity of SGR has been suggested against hepatocarcinomas [20,21], and a glycoprotein, SGF2, which is isolated from SGR has been reported to have antiproliferative effects on MCF-7 breast cancer cells [22]. However, these reports have focused on the antiproliferative or apoptotic effects of SGR on cancer cell lines. In our study, we estimated the regulatory function and molecular mechanisms of SGR, particularly in regard to adhesion and migration of prostate cancer cells. In addition, we estimated the single components of WESGR through HPLC-MS/MS analysis.

## 2. Results

### 2.1. Collagen-Dependent Adhesion and Migration of PC3 and LNCaP Prostate Cancer Cells are Effectively Attenuated by Treatment with Nontoxicological Levels of WESGR

**Figure 1:**
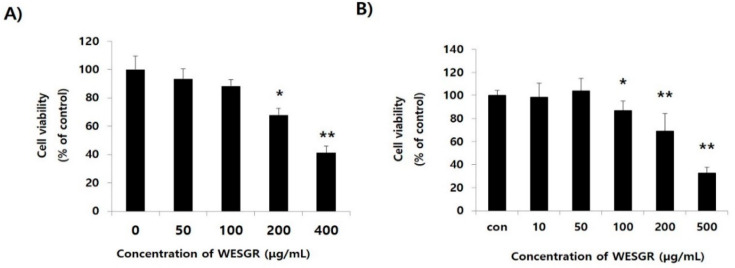
Cytotoxicity of water extract of *Smilax glabra* Roxb. (WESGR) on PC3 (**A**) and LNCaP (**B**) prostate cancer cells. WESGR was administered to PC3 and LNCaP cells for 24 h, and the viability was estimated with a WST-1 Assay Kit. All values are expressed as the mean ± standard deviation (SD) of three wells. *and ** indicate significant differences compared to the control; * = *p* < 0.050 and ** = *p* < 0.001.

The nontoxicological levels of WESGR on PC3 and LNCaP prostate cancer cells were measured with a WST-1 assay kit which could detect metabolically active cells in the culture. As shown in Figure 1, WESGR up to the concentration of 100 and 50 μg/mL did not show cytotoxicity to PC3 and LNCaP cells, respectively; however, the viability significantly decreased when 200 and 100 μg/mL of WESGR was added to the PC3 and LNCaP cells, respectively. In addition, the cytotoxic potential of WESGR was further evaluated during 96 h of incubation. WESGR (100 μg/mL for PC3 and 50 μg/mL for LNCaP) did not affect the viability of PC3 and LNCaP cells until 72 h of incubation (Supplementary Figure S1). Based on these results, we investigated the effects of nontoxicological levels of WESGR (25–100 μg/mL for PC3 cells and 25–50 μg/mL for LNCaP cells) on cell adhesion and migration.

**Figure 2:**
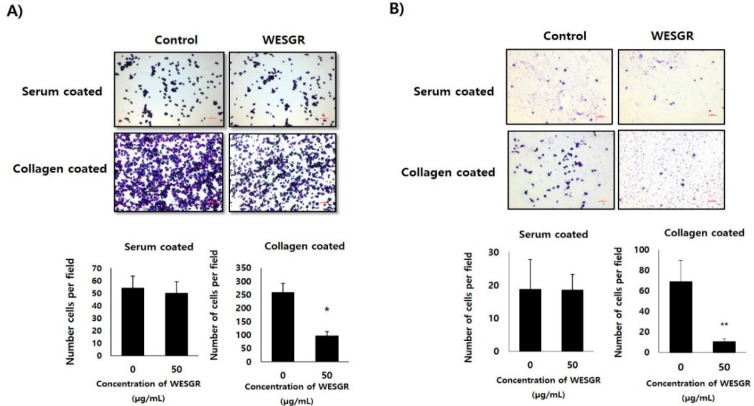
Collagen-mediated adhesion in PC3 (**A**) and LNCaP (**B**) cells was inhibited by WESGR. PC3 and LNCaP cells were pretreated with and without WESGR (50 μg/mL) and added to a serum or collagen I-coated plate. After washing with phosphate-buffered saline (PBS), the attached PC3 and LNCaP cells were fixed, stained with 5% crystal violet, and counted. The data shown are representative of at least three independent experiments. Error bars indicate the SD of the mean. Bar = 200 μm. *and ** indicate significant differences compared to the control; * = *p* < 0.050 and ** = *p* < 0.001.

Since it has been reported that, among the ECM proteins, the most abundant migration of PC3 and LNCaP cells is toward collagen [12], we thought that if WESGR attenuates interaction between collagen and PC3 and LNCaP cells, it could be developed as an anti-prostate-cancer drug for attenuating events in the prostate cancer tumorigenesis and metastasis, such as adhesion or migration. Therefore, we first examined whether the nontoxicological levels of WESGR could attenuate collagen-dependent adhesion. Figure 2 shows that nontoxicological levels of WESGR did not affect PC3 and LNCaP cell adhesion on a serum-coated plate. However, more than 50% of PC3 and LNCaP cells became unable to adhere to a collagen-coated bottom region after treatment with nontoxicological levels of WESGR (50 μg/mL). These results suggest that the use of nontoxicological levels of WESGR could be an effective way to attenuate the progression of prostate cancer during particular stages, such as prostate cancer cell adhesion to collagen.

**Figure 3:**
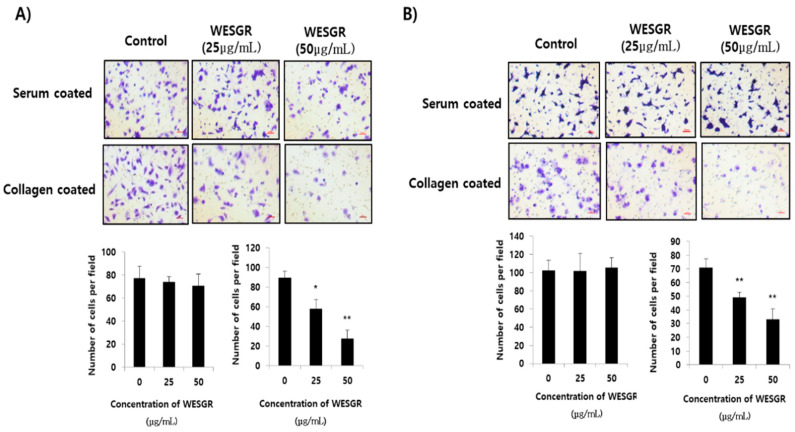
Collagen-dependent migration of PC3 (**A**) and LNCaP (**B**) cells was attenuated by WESGR. PC3 (A) and LNCaP (B) cells that were untreated and pretreated with WESGR were loaded to the upper side of the membrane. Cells that migrated to the bottom area that was coated with either 20% serum or collagen I were stained with 5% crystal violet and counted. Error bars represent the standard deviation (SD) of the mean. The bars represent 200 μm of 200× magnification. * = *p* < 0.050, ** = *p* < 0.001, compared with control (untreated).

We next investigated whether WESGR could attenuate the migration of PC3 and LNCaP cells toward collagen. PC3 and LNCaP cells that had been pretreated with nontoxicological levels of WESGR and those cells that were not treated were loaded to the upper side of a Transwell chamber and incubated in a CO_2_ incubator. Figure 3 shows that nontoxicological levels of WESGR did not lessen the migration of PC3 and LNCaP cells from the upper side of the membrane to the bottom, which was coated with serum. However, the migration of PC3 and LNCaP cells to the collagen-coated bottom area was dose dependently attenuated by WESGR treatments. These results suggest that the use of nontoxicological levels of WESGR could be an effective method of attenuating the progression of prostate cancer at particular stages, such as the migration of prostate cancer cells toward collagen. In addition, we also found that administration of WESGR inhibited the migration of PC3 cells on collagen-coated plate but not on the serum-coated plate by wound-healing analysis (Supplementary Figure S2). These results suggest that impaired cell transmigration by WESGR treatment results from impaired cell motility as well as mechanical elasticity.

### 2.2. Collagen-Dependent Expression of β1 Integrin in PC3 and LNCaP Prostate Cancer Cells is Inhibited by Treatment with WESGR

**Figure 4:**
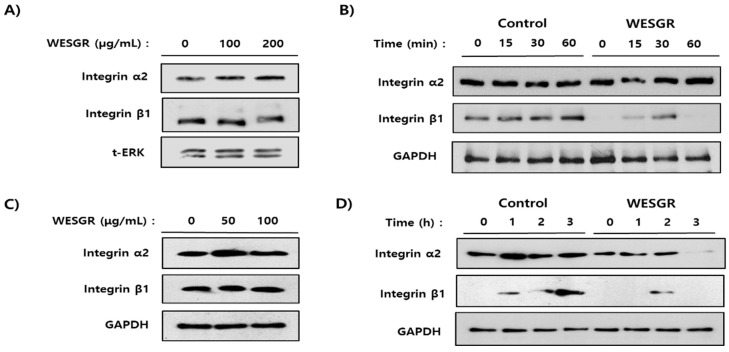
The expression of α2 and β1 integrin of PC3 (**A** and **B**) and LNCaP (**C** and **D**) was estimated by Western blotting. Administration of WESGR to PC3 (**A**) and LNCaP (**C**) cells did not change the expression of α2β1 integrin. PC3 (**B**) and LNCaP (**D**) cells that were untreated and pretreated for 30 min (PC3 cells) or 2 h (LNCaP cells) with WESGR (100 μg/mL for PC3 and 50 μg/mL for LNCaP) were seeded on a collagen-coated plate and harvested at the indicated times. The data shown are representative of at least three independent experiments.

After confirming that nontoxicological levels of WESGR could attenuate PC3 and LNCaP cells migration, but only toward collagen, we investigated whether nontoxicological levels of WESGR could attenuate direct interactions between collagen and prostate cancer cells (PC3 and LNCaP). As shown in Figure 2, nontoxicological levels of WESGR significantly inhibited collagen-dependent PC3 and LNCaP cell adhesion. Based on this result, we next examined the effect of WESGR on the expression of α2β1 integrin, which is a known receptor for collagen in PC3 and LNCaP cells. Ectopic administration of WESGR up to a concentration of 200 and 100 μg/mL did not affect the expression of α2β1 integrin on already attached PC3 and LNCaP cells, respectively (Figure 4A,C). Interestingly, pretreatment with WESGR (100 μg/mL) significantly attenuated the expression of β1 integrin but did not reduce α2 integrin expression during the process of PC3 cell adhesion to a collagen-coated plate (Figure 4B). In addition, the expression of α2 integrin on LNCaP cells at 3 h after seeding to collagen-coated plate was decreased by the pretreatment of WESGR (50 μg/mL), and the expression of β1 integrin was attenuated by pretreatment of WESGR (50 μg/mL) during the process of LNCaP cells adhesion to collagen-coated plate (Figure 4D). The expression of β1 integrin seems to gradually increase in the process of adhesion (Figure 4B,D) and once cells attached to matrix, the expression of β1 integrin seems to be saturated (Figure 4C). Taken together, these data suggest that administration of WESGR in the process of adhesion could inhibit the direct interaction between prostate cancer cells (PC3 and LNCaP) and collagen which is responsible for induction of β1 integrin expression.

### 2.3. Collagen-Dependent FAK Signaling of PC3 and LNCaP Prostate Cancer Cells is Attenuated by Treatment with WESGR

**Figure 5:**
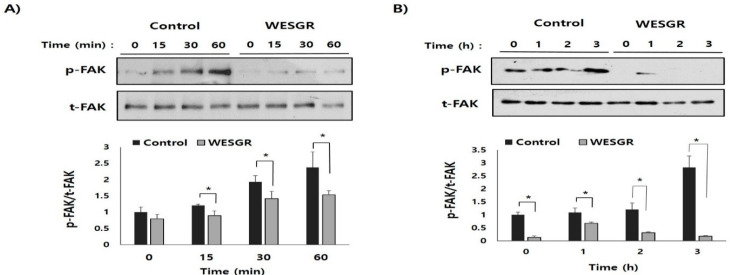
Collagen-dependent activation/phosphorylation of FAK was attenuated by WESGR. PC3 (**A**) and LNCaP (**B**) cells that were untreated and pretreated with WESGR were seeded on collagen-coated plates. Cells were harvested at the indicated time points, and the phosphorylation of FAK and total levels of FAK protein were measured using Western blotting. The activation rate of FAK (p-FAK/t-FAK) was estimated using the ImageJ program. *p < 0.05 when compared with control.

To further investigate the detailed molecular mechanisms of WESGR-mediated regulation of migration toward and adhesion to collagen, we investigated whether the collagen-mediated activation of FAK, which has an important role in integrin-dependent migration and adhesion, is regulated by the treatment of PC3 and LNCaP cells with WESGR. As shown in Figure 5, with PC3 and LNCaP cells placed on a membrane with a collagen-coated plate, the activation/phosphorylation of FAK gradually increased in the control (untreated) PC3 and LNCaP cells; however, the PC3 and LNCaP cells that had been treated with nontoxicological levels of WESGR showed attenuated phosphorylation of FAK during the adhesion process to collagen. These results indicate that integrin-dependent signaling could be effectively inhibited by WESGR to slow PC3 and LNCaP cells adhesion to collagen-rich ECM regions.

### 2.4. Single Components Were Analyzed by HPLC-MS/MS

**Figure 6:**
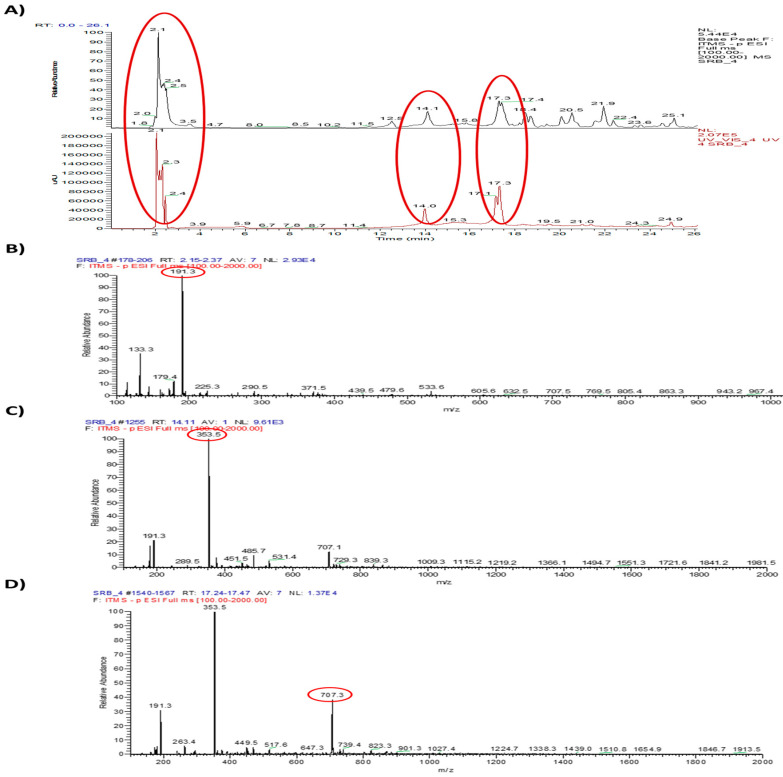
Single constituent of WESGR was analyzed by HPLC-MS/MS. The molecular weights of three major peaks (t_R_ 2.1, 14.1, and 17.3 min) (**A**) were estimated as 191.3 (**B**), 363.5 (**C**), and 707.3 (**D**) Da, respectively.

To estimate the functional single components of WESGR, it was firstly separated by HPLC (Figure 6A) and three major peaks (tR 2.1, 14.1, and 17.3 min) were further analyzed by mass spectrometry (Figure 6B–D). Main peak of each results of mass was selected and further characterized by MS/MS analysis. Observed mass of three major peak of WESRG and the results of LC-MA/MS were showed in Table 1. By referencing to the reported data, it was found that 5-*O*-caffeoylquinic acid, 4-*O*-caffeoylquinic acid, and 3-*O*-caffeoylquinic acid may be major constituents of WESGR.

### 2.5. 5-O-Caffeoylquinic Acid is Determined as a Functional Single Component of WESGR

**Figure 7:**
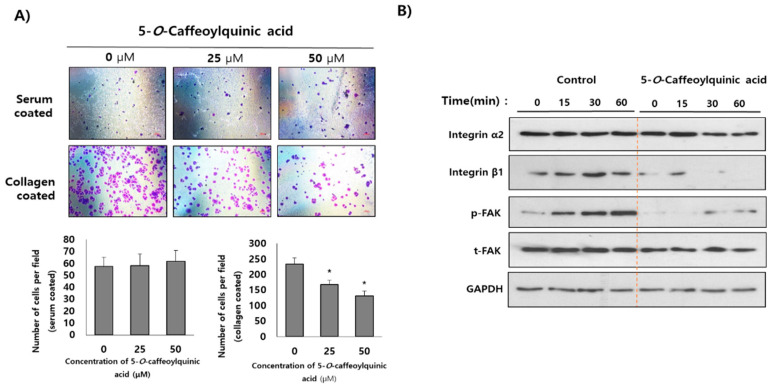
Collagen-mediated adhesion in PC3 cells was inhibited by 5-*O*-caffeoylquinic acid. PC3 cells were pretreated (3 h) with and without 5-*O*-caffeoylquinic acid (25 and 50 μM) and added to a serum or collagen I-coated plate. After washing with phosphate-buffered saline (PBS), the attached PC3 cells were fixed, stained with 5% crystal violet, and counted (**A**). The number of adherent cells were counted of four different areas and tabulated. PC3 cells which were treated with and without 5-caffeoylquinic acid (50 μM) were seeded on a collagen-coated plate and harvested at the indicated times. The expression of α2 integrin, β1 integrin phospho-FAK, total FAK, and GAPDH was estimated by Western blotting (**B**). Error bars indicate the standard deviation (SD) of the mean. Bar = 200 μm. *p < 0.05 when compared with control.

Among the several single components of WESGR, antiadhesive property of 5-*O*-caffeoylquinic was investigated. As shown in Figure 7A, pretreatment of nontoxicological levels of 5-*O*-caffeoylquinic acid (25 and 50 μM) attenuated the collagen-mediated PC3 cell adhesion but did not affect PC3 cell adhesion on a serum-coated plate. Furthermore, pretreatment of nontoxicological levels of 5-*O*-caffeoylquinic acid attenuated the expression of β1 integrin but did not inhibit α2 integrin expression during the process of PC3 cell adhesion resulted in impaired phosphorylation of FAK (Figure 7B). These results suggest that 5-*O*-caffeoylquinic acid is a major functional single component of WESGR which accounts for antiprostate cancer effects of WESGR.

## 3. Discussion

For the development of effective antiprostate cancer agents that are able to attenuate metastatic steps, our group screened potential natural agents that might attenuate the interaction between collagen and prostate cancer cells and also investigated the potential target molecules responsible for their inhibition. Water extracts of *Gleditsia sinensis* thorns (WEGST) was defined in our previous study [28] as a potential antiprostate cancer agent that can attenuate the adhesion of prostate cancer cells to collagen, and the antiprostate cancer activity of water extracts of *Smilax glabra* Roxb. (WESGR) was characterized in this study.

Prostate cancer causes the second highest mortality level from cancer in men, and it has been reported that prostate cancer metastasis leads to bony lesions can be easily found in men who die from prostate cancer [29]. Although several anticancer drugs such as paclitaxel, vinblastine, and docetaxel, which aim to inhibit cell proliferation by attenuating mitotic spindle formation, have been developed, resistance to these drugs may occur in a portion of prostate cancer cells, resulting in the development of mCRPC [30]. Furthermore, there is a limitation to using high doses of anticancer drugs to reach lethal toxicity because such doses could affect the viability of normal cells and result in severe side effects. Therefore, it seems very difficult to completely eliminate prostate cancer with only antiprostate cancer drugs that target attenuation of proliferation.

Because adhesion and migration are among the critical steps of prostate cancer progression, we hypothesized that antiprostate cancer agents that target specific steps, such as adhesion and migration, might be an effective alternative treatment to cure patients suffering from prostate cancer as well as mCRPC. So, we investigated whether nontoxicological levels of WESGR could suppress collagen-mediated adhesion and migration of PC3 and LNCaP prostate cancer cells. Interestingly, pretreatment with WESGR (25 and 50 μg/mL, respectively) dose dependently inhibited PC3 and LNCaP cell migration toward collagen but not to serum.

Integrin is a well-known heterodimeric receptor for ECM proteins and is reported to play important roles in cell migration and attachment. Among the several integrins, α2β1 integrin, which is a receptor for collagen, is abundantly expressed in prostate cancer cells [11], and the invasiveness of PC3 cells could be attenuated by administration of α2 and β1 neutralizing antibodies, but not α1 and α6 neutralizing antibodies [12], indicating that prostate cancer cells may primarily use α2β1 integrin during migration and adhesion. Therefore, α2β1 integrin could be a potential molecular target for inhibiting the progression of prostate cancer. After finding that WESGR could significantly attenuate collagen-mediated migration and adhesion, we investigated whether WESGR might inhibit the expression of α2β1 integrin. Interestingly, ectopic administration of WESGR did not affect the expression of α2β1 integrin on PC3 and LNCaP cells. However, pretreatment with WESGR inhibited collagen-induced expression of β1 integrin when PC3 and LNCaP cells were placed on a collagen-coated plate, suggesting that WESGR may attenuate the collagen-mediated intracellular signaling for activating β1 integrin expression during the adhesion process. If the expression of β1 integrin is downregulated, the heterodimerization of α2β1 should be disrupted, and subsequently, integrin-mediated intracellular signaling, such as with FAK, should be inactivated. Interestingly, pretreatment with WESGR attenuated the collagen-mediated expression of β1 integrin as well as the phosphorylation of FAK. In addition, we also found that pretreatment of WESGR inhibited the collagen-mediated actin formation on PC3 and LNCaP cells which is important in lamellipodium formation (Supplementary Figure S3). These data suggested that WESGR could effectively inhibit the collagen-mediated prostate cancer cells migration and adhesion.

In our previous study, WEGST was defined as a potential antiprostate cancer agent that can inhibit the adhesion of prostate cancer cells to collagen through attenuation of the expression of α2 integrin, but not that of β1 integrin [12]. Interestingly, WESGR showed a similar activity but had different target specificity. WESGR attenuated the collagen-mediated migration and adhesion, but the major target molecule of WESGR was β1 integrin. We think that different target specificity of both WEGST and WESGR may come from different active principles of these extracts. Lupine acid, ethyl gallate, and stigmasterol were suggested as active component of WEGST [12,31,32,33], however caffeoylquinic acids were proposed as major components of WESGR in this study. These results suggest that if a new agent was developed that comprised both WEGST and WESGR, it might inhibit the migration and adhesion of prostate cancer cells more effectively than a single treatment with either WEGST or WESGR because it could attenuate the expression of both α2 and β1 integrin during prostate cancer cell migration toward and adhesion to the ECM. A functional analysis of a new agent with both WEGST and WESGR will be performed in our further study.

To determine the major constituents of WESGR, HPLC/MS/MS analysis was performed. By referencing to the reported data, 5-*O*-caffeoylquinic acid, 4-*O*-caffeoylquinic acid, and 3-*O*-caffeoylquinic acid may be major constituents of WESGR. Caffeoylquinic acids are composed of quinic acid core and one or more caffeoyl groups. The proliferation of cancer cells seems to be attenuated by caffeoylquinic acids directly. For examples, it was reported that 5-*O*-caffeoylquinic acid has antiproliferative effects on MBA-MB-231 breast cancer cells through modulating the Ras-dependent signaling [25], and caffeoylquinic acid derivatives seems to have cell cycle arrest and apoptosis induction properties in gastric adenocarcinoma (AGS) cells [34]. In addition, it was reported that mitogen-stimulated invasion but not proliferation of non-small cell lung cancer (NSCLC) could be attenuated by administration of 5-*O*-caffeoylquinic acid [35]. Interestingly, we found that pretreatment of 5-*O*-caffeoylquinic acid effectively attenuated collagen-mediated PC3 cell adhesion. In addition, collagen-induced β1 integrin expression and FAK activation were also attenuated by pretreatment of 5-*O*-caffeoylquinic acid. Therefore, taken together, there is possibility that 5-*O*-caffeoylquinic acid could be the active component which accounts for anti-tumor effects of WESGR.

In conclusion, our results strongly suggest that nontoxicological levels of WESGR could be used for attenuating the progression of particular steps in prostate cancer metastasis, such as migration and adhesion, through restricting collagen-mediated β1 integrin expression. More detailed functional analysis of 5-*O*-caffeoylquinic acid on the progress of prostate cancer by using in vivo animal model will be helpful for understanding the more detailed mechanism of WESGR-mediated antiprostate cancer effects and will be performed in our future study.

## 4. Materials and Methods

### 4.1. Preparation of WESGR

*Smilax glabra* Roxb. (SGR) was purchased from Kyungdong market in Seoul, South Korea and 200 g of SGR was extracted with 2 L of hot water. The supernatant was harvested, filtered, and concentrated using rotary evaporation system (Heidolph Instruments GmbH & Co., Schwabach, Germany). Final concentrates were lyophilized and kept at −80 °C in a refrigerator until use. The yield of the dried extract was approximately 6.8 g/L.

### 4.2. Cytotoxicity Assay of WESGR

PC3 and LNCaP cells obtained from the American Type Culture Collection (ATCC; Manassas, VA, USA) were seeded in a 96-well plate (5 × 10^3^ cells/well for PC3 cells and 4 × 10^4^ cells/well for LNCaP cells) and cultured in Roswell Park Memorial Institute (RPMI) 1640 medium supplemented with 10% fetal bovine serum (FBS) (HyClone Laboratories, Logan, UT, USA) and 1% penicillin–streptomycin solution (BioWhittaker Inc., Walkersville, MD, USA) for 24 h. After washing with RPMI 1640 medium, WESGR was added to each well at various concentrations (50–400 μg/mL for PC3 cells, and 10–500 μg/mL for LNCaP cells) and incubated at 37 °C in a CO_2_ incubator for 24 h. Subsequently, 2-(4-Iodophenyl)-3-(4-nitrophenyl)-5-(2,4-disulfophenyl)-2H-tetrazolium, monosodium salt (WST-1) solution (Dozen, Seoul, Korea) was added to each well, and the cells were incubated for a further 2 h. The absorbance was estimated at 450 nm with a microplate reader (Model 680 Microplate Reader, Bio-Rad, Hercules, CA, USA).

### 4.3. Cell Adhesion Assay

Prostate cancer cells (PC3 and LNCaP) were pretreated with and without WESGR (50 μg/mL) for 6 h, and then, those were loaded on collagen I (10 μg/mL) or 20% FBS-coated 96-well plates (2 × 10^5^ cells/well for PC3 cells and 1 × 10^5^ cells/well for LNCaP cells). After further incubation for 15 min (PC3 cells) and 3 h (LNCaP cells) at room temperature, the plates were washed once with PBS. The remaining cells were fixed with paraformaldehyde solution (4% w/v) and then stained with crystal violet solution (5% w/v). The number of adhered cells was then counted under a microscope.

### 4.4. Collagen Against Migration Assay

The antimigratory effects of WESGR on the collagen-dependent migration of PC3 and LNCaP cells were measured using Transwell Permeable Supports (BD Biosciences, San Jose, A, USA). The bottom area of the Transwell membrane was coated with serum (20% w/v) or collagen I (10 μg/mL, Sigma-Aldrich Co., St. Louis, MO, USA) overnight in PBS at 4 °C. The PC3 cells (5 × 10^4^ cells/well) and LNCaP cells (2 × 10^5^ cells/well), respectively, were pretreated with 25 and 50 μg/mL of WESGR for 6 h and then added to the upper side of a Transwell chamber (8 μm pore size). The PC3 cells were further incubated for 6 h (serum-coated Transwell) or 2 h (collagen-coated Transwell) at 37 °C in a CO_2_ incubator, whereas the LNCaP cells were further incubated for 18 h (serum-coated Transwell) or 6 h (collagen-coated Transwell) at 37 °C in a CO_2_ incubator. Any PC3 and LNCaP cells that migrated to the bottom layer were fixed with paraformaldehyde (4% w/v) and then stained with crystal violet (0.5% w/v).

### 4.5. Analysis of Collagen for Expression of α2β1 Integrin and Focal Adhesion Kinase (FAK)

About 70% confluent PC3 and LNCaP cells were washed twice with PBS and separated into single cells by treatment with a trypsin/EDTA solution (HyClone Laboratories, Logan, UT, USA). The activity of trypsin was deactivated by treatment with a trypsin inhibitor from glycine max soybean (Sigma-Aldrich Co., St. Louis, MO, USA). PC3 and LNCaP cells were either not treated or were pretreated with WEGST (100 μg/mL for PC3 and 50 μg/mL for LNCaP) for 30 min (PC3 cells) or 2 h (LNCaP cells) and then seeded on collagen I (10 μg/mL)-coated plates. Cells were harvested at various time points, as indicated, and total proteins were solubilized with a lysis buffer composed of 20 mM Tris-HCl (pH 7.4), Nonidet P-40 (1% w/v), 150 mM NaCl, 5 mM EDTA, 10 mM NaF, 5 mM sodium pyrophosphate, 1 mM sodium orthovanadate, 10 mM β-glycerophosphate, 1 mM phenylmethylsulfonyl fluoride, and a protease inhibitor mixture (Sigma-Aldrich Co., St. Louis, MO, USA). Cell lysates were separated by sodium dodecyl sulfate polyacrylamide gel electrophoresis (SDS-PAGE) and transferred to polyvinylidene difluoride (PVDF) membranes. The membranes were incubated separately with an antiphospho-FAK antibody (BD Biosciences, San Jose, CA, USA), mouse anti-FAK antibody (BD Biosciences, San Jose, CA, USA), rabbit anti-α2 integrin antibody (Merck Millipore, Gibbstown, NJ, USA), anti-β1 integrin antibody (sc-374429) (Santa Cruz, CA, USA), and mouse anti-β-actin antibody (Santa Cruz, CA, USA), followed by horseradish peroxidase (HRP)-conjugated antirabbit immunoglobulin G (IgG). ECL reagents (Bio-Rad Co., Hercules, CA, USA) were used to activate signals.

### 4.6. HPLC-MS/MS Analysis

HPLC-MS/MS analysis was performed to determine the major constituents of WESGR. The analysis was carried out on an Ultimate 3000 RS system (Thermo Fisher Scientific, San Jose, CA, USA) coupled with LTQ Ion Trap Mass Spectrometer (Thermo Fisher Scientific, San Jose, CA, USA). WESRG was separated on a Phenomenex Kinetex C18 column (150 mm, 2.10 mm, 1.7 um, Phenomenex Inc., USA) by using a flow rate of 0.2 mL/min at 40 °C. The mobile phase of eluent A was aqueous formic acid solution, 0.1% v/v and that of eluent B was methanol with formic acid, 0.1%, v/v. A gradient program was used for elution: 0–30 min, A from 99% to 0%, and B from 1% to 100%. Ion Trap MS and spray chamber conditions were capillary temperature of 300 °C and source voltage of 3.5 kV.

### 4.7. Statistical Analysis

Statistical analysis was performed using Prism 5 software (GraphPad Software, Inc., San Diego, CA, USA). The statistical significance between two samples was analyzed using unpaired student’s *t*-test. The results are presented as mean ± standard deviation (SD). A *p* value of < 0.05 was considered to be significant.

## Figures and Tables

**Table 1:** Major constituents of water extract of *Smilax glabra* Roxb. (WESGR) by LC/MS/MS.

Number	t_R_ (min)	λ_max_ (nm)	Observed Mass	Main Peak	LC-MS^2^ of Main Peak	Compounds (MW)	Ref.
1	2.1	340	133.3, 179.4, 191.3, 225.3, 290.5, 371.5, 439.5, 479.6, 533.6, 605.6, 632.5, 707.5, 769.5, 805.4, 863.3, 943.2, 967.4	191.3	58.9, 70.9, 83.0, 84.9, 92.9, 98.9, 109.0, 110.0, 127.0, 137.0, 153.0, 155.0, 171.0, 173.0, 174.0, 191.1	N.D.	–
2	14.1	340	191.3, 289.5, 353.5, 451.5, 485.7, 531.4, 707.1, 729.3, 839.3, 1009.3, 1115.2, 1219.2, 1366.1, 1494.7, 1551.3, 1721.6, 1841.2, 1981.5	353.5	135.0, 160.9, 172.9, 191.0, 192.1, 197.1, 217.2	Catechin (289.5)	[23,24]
						Epicatechin (289.5)	[23,24]
						5-*O*-Caffeoylquinic acid (353.086)	[25]
						4-*O*-Caffeoylquinic acid (353,086)	[26,27]
						3-*O*-Caffeoylquinic acid (353.086)	[26,27]
						Cinchonain Ib (451.5)	[23,24]
3	17.3	340	191.3, 263.4, 353.5, 449.5, 517.6, 647.3, 707.3, 739.4, 823.3, 901.3, 1027.4, 1224.7, 1338.3, 1439.0, 1510.8, 1654.9, 1846.7, 1913.5	707.1	307.0, 351.1, 353.1, 353.7, 452.3, 514.3, 555.2, 617.1, 658.1, 689.3	Neoastilbin (449.5)	[23,24]
						Astilbin (449.5)	[23,24]

N.D: not determined.

## Supplementary Materials

The following are available online. Figure S1: Cytotoxicity of WESGR or 5-*O*-caffeoylquinic acid on PC3 and LNCaP prostate cancer cells: Figure S2: After treatement, PC3 cells were subjected to the scratch wound assay. Figure S3: Treatment of WESGR attenuated collagen induced actin formation of PC3 and LNCaP cells.

## References

[1] Daniyal M., Siddiqui Z.A., Akram M., Asif H., Sultana S., Khan A. (2014). Epidemiology, Etiology, Diagnosis and Treatment of Prostate Cancer. Asian Pac. J. Cancer Prev..

[2] E Fleshner N., Evans A., Chadwick K., Lawrentschuk N., Zlotta A. (2010). Clinical significance of the positive surgical margin based upon location, grade, and stage. Urol. Oncol. Semin. Orig. Investig..

[3] Dellis A., Zagouri F., Liontos M., Mitropoulos D., Bamias A., Papatsoris A.G. (2019). Management of advanced prostate cancer: A systematic review of existing guidelines and recommendations. Cancer Treat. Rev..

[4] Rycaj K., Tang D.G. (2017). Molecular determinants of prostate cancer metastasis. Oncotarget.

[5] Hynes R.O. (1992). Integrins: Versatility, modulation, and signaling in cell adhesion. Cell.

[6] Rosales C., O’Brien V., Kornberg L., Juliano R. (1995). Signal transduction by cell adhesion receptors. Biochim. et Biophys. Acta (BBA) - Bioenerg..

[7] E Hughes P., Renshaw M.W., Pfaff M., Forsyth J., Keivens V.M., Schwartz M.A., Ginsberg M.H. (1997). Suppression of Integrin Activation: A Novel Function of a Ras/Raf-Initiated MAP Kinase Pathway. Cell.

[8] Desgrosellier J.S., Cheresh D.A. (2010). Integrins in cancer: biological implications and therapeutic opportunities. Nat. Rev. Cancer.

[9] Mitchell K., Svenson K.B., Longmate W.M., Gkirtzimanaki K., Sadej R., Wang X., Zhao J., Eliopoulos A., Berditchevski F., DiPersio C.M. (2010). Suppression of integrin alpha3beta1 in breast cancer cells reduces cyclooxygenase-2 gene expression and inhibits tumorigenesis, invasion, and cross-talk to endothelial cells. Cancer Res..

[10] Saito Y., Sekine W., Sano R., Komatsu S., Mizuno H., Katabami K., Shimada K., Oku T., Tsuji T. (2010). Potentiation of cell invasion and matrix metalloproteinase production by alpha3beta1 integrin-mediated adhesion of gastric carcinoma cells to laminin-5. Clin. Exp. Metastasis.

[11] Bonaccorsi L., Carloni V., Muratori M., Salvadori A., Giannini A., Carini M., Serio M., Forti G., Baldi E. (2000). Androgen receptor expression in prostate carcinoma cells suppresses alpha6beta4 integrin-mediated invasive phenotype. Endocrinology.

[12] Lee S.H., Hatakeyama S., Yu S.Y., Bao X., Ohyama C., Khoo K.H., Fukuda M.N., Fukuda M. (2009). Core3 O-glycan synthase suppresses tumor formation and metastasis of prostate carcinoma PC3 and LNCaP cells through down-regulation of alpha2beta1 integrin complex. J. Biol. Chem..

[13] Slambrouck V., Van Slambrouck S., Jenkins A.R., Romero A.E., Steelant W.F. (2009). Reorganization of the integrin α2 subunit controls cell adhesion and cancer cell invasion in prostate cancer. Int. J. Oncol..

[14] Bonkhoff H., Stein U., Remberger K. (1993). Differential expression of α6 and α2 very late antigen integrins in the normal, hyperplastic, and neoplastic prostate: Simultaneous demonstration of cell surface receptors and their extracellular ligands. Hum. Pathol..

[15] Fukunaga T., Miura T., Furuta K., Kato A. (1997). Hypoglycemic Effect of the Rhizomes of Smilax glabra in Normal and Diabetic Mice. Boil. Pharm. Bull..

[16] Xia D., Yu X., Liao S., Shao Q., Mou H., Ma W. (2010). Protective effect of Smilax glabra extract against lead-induced oxidative stress in rats. J. Ethnopharmacol..

[17] Ooi L.S.M., Sun S.S.M., Wang H., Ooi V.E.C. (2004). New Mannose-Binding Lectin Isolated from the Rhizome of SarsaparillaSmilax glabraRoxb. (Liliaceae). J. Agric. Food Chem..

[18] Galhena B.P., Samarakoon S.R., Thabrew M.I., Weerasinghe G.A.K., Thammitiyagodage M.G., Ratnasooriya W.D., Tennekoon K.H. (2012). Anti-Inflammatory Activity Is a Possible Mechanism by Which the Polyherbal Formulation Comprised of Nigella sativa (Seeds), Hemidesmus indicus (Root), and Smilax glabra (Rhizome) Mediates Its Antihepatocarcinogenic Effects. Evidence-Based Complement. Altern. Med..

[19] Jiang J., Xu Q. (2003). Immunomodulatory activity of the aqueous extract from rhizome of Smilax glabra in the later phase of adjuvant-induced arthritis in rats. J. Ethnopharmacol..

[20] Sa F., Gao J.-L., Fung K.-P., Zheng Y., Lee S.M.Y., Wang Y.-T. (2008). Anti-proliferative and pro-apoptotic effect of Smilax glabra Roxb. extract on hepatoma cell lines. Chem. Interactions.

[21] Samarakoon S.R., Thabrew I., Galhena B.P., Tennekoon K.H. (2012). Modulation of apoptosis in human hepatocellular carcinoma (HepG2 cells) by a standardized herbal decoction of Nigella sativa seeds, Hemidesmus indicus roots and Smilax glabra rhizomes with anti- hepatocarcinogenic effects. BMC Complement. Altern. Med..

[22] Ooi L.S.M., Wong E.Y.L., Chiu L.C.M., Sun S.S.M., Ooi V.E.C. (2008). Antiviral and Anti-proliferative Glycoproteins from the Rhizome of Smilax glabra Roxb (Liliaceae). Am. J. Chin. Med..

[23] Lu C.-L., Zhu W., Wang M., Xu X.-J., Lu C.-J. (2014). Antioxidant and Anti-Inflammatory Activities of Phenolic-Enriched Extracts of Smilax glabra. Evidence-Based Complement. Altern. Med..

[24] She T., Qu L., Wang L., Yang X., Xu S., Feng J., Gao Y., Zhao C., Han Y., Cai S. (2015). Sarsaparilla (Smilax Glabra Rhizome) Extract Inhibits Cancer Cell Growth by S Phase Arrest, Apoptosis, and Autophagy via Redox-Dependent ERK1/2 Pathway. Cancer Prev. Res..

[25] Palmioli A., Ciaramelli C., Tisi R., Spinelli M., De Sanctis G., Sacco E., Airoldi C. (2017). Natural Compounds in Cancer Prevention: Effects of Coffee Extracts and Their Main Polyphenolic Component, 5-O -Caffeoylquinic Acid, on Oncogenic Ras Proteins. Chem. Asian J..

[26] Choi Y.H., Kim H.K., Linthorst H.J., Hollander J.G., Lefeber A.W., Erkelens C., Nuzillard J.M., Verpoorte R. (2006). NMR metabolomics to revisit the tobacco mosaic virus infection in Nicotiana tabacum leaves. J. Nat. Prod..

[27] Nakatani N., Kayano S., Kikuzaki H., Sumino K., Katagiri K., Mitani T. (2000). Identification, quantitative determination, and antioxidative activities of chlorogenic acid isomers in prune (*Prunus domestica* L.). J. Agric. Food Chem..

[28] Ryu S., Park K.M., Lee S.H. (2016). Gleditsia sinensis Thorn Attenuates the Collagen-Based Migration of PC3 Prostate Cancer Cells through the Suppression of alpha2beta1 Integrin Expression. Int. J. Mol. Sci..

[29] Bubendorf L., Schöpfer A., Wagner U., Sauter G., Moch H., Willi N., Gasser T.C., Mihatsch M.J. (2000). Metastatic patterns of prostate cancer: an autopsy study of 1589 patients. Hum. Pathol..

[30] Seruga B., Tannock I.F. (2011). Chemotherapy-Based Treatment for Castration-Resistant Prostate Cancer. J. Clin. Oncol..

[31] Cui H.-X., Wang M., Yuan J.-X., Liu J.-C. (2015). [Effect of ethyl gallate on invasion abilities and its mechanism of breast cancer MDA-MB-231 cells]. Yao xue xue bao = Acta Pharm. Sin..

[32] Lim J.-C., Park J.H., Buděšínský M., Kasal A., Han Y.-H., Koo B.-S., Lee S.-I., Lee D.-U. (2005). Antimutagenic constituents from the thorns of Gleditsia sinensis. Chem. Pharm. Bull..

[33] Tanaka R., Kinouchi Y., Wada S.-I., Tokuda H. (2004). Potential Anti-Tumor Promoting Activity of Lupane-Type Triterpenoids from the Stem Bark ofGlochidion zeylanicumandPhyllanthus flexuosus. Planta Med..

[34] Jafari N., Zargar S.J., Delnavazi M.R., Yassa N. (2018). Cell Cycle Arrest and Apoptosis Induction of Phloroacetophenone Glycosides and Caffeoylquinic Acid Derivatives in Gastric Adenocarcinoma (AGS) Cells. Anti-Cancer Agents Med. Chem..

[35] In J.-K., Kim J.-K., Oh J.S., Seo D.-W. (2016). 5-Caffeoylquinic acid inhibits invasion of non-small cell lung cancer cells through the inactivation of p70S6K and Akt activity: Involvement of p53 in differential regulation of signaling pathways. Int. J. Oncol..

