# Supplementary material for: Smilax glabra Roxb. Inhibits Collagen Induced Adhesion and Migration of PC3 and LNCaP Prostate Cancer Cells through the Inhibition of Beta 1 Integrin Expression
Source: Molecules. 2020 Jun 30;25(13):3006. doi: 10.3390/molecules25133006 (PMC7411785; doi:10.3390/molecules25133006)
Supplement: Supplementary file 1 [file molecules-25-03006-s001.pdf]

molecules

MDPI

# 1. Supplementary Results

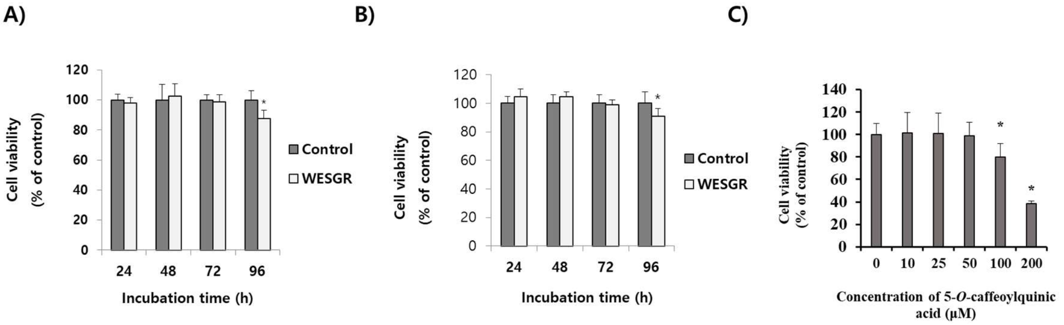


**Supplementary figure 1.** Cytotoxicity of WESGR or 5-O-caffeoylquinic acid on PC3 (A, C) and LNCaP (B) prostate cancer cells. WESGR (100 μg/mL for PC3 and 50 μg/mL for LNCaP) did not affects the viability of PC3 and LNCaP cells until 72 h of incubation. Various concentrations of 5-*O*-caffeoylquinic acid was administered to PC3 cells for 24 h and the viability was estimated with a WST-1 assay kit. All values are expressed as the mean ± standard deviation (SD) of three wells. * indicate significant differences compared to the control (untreated); * = $p < 0.05$.

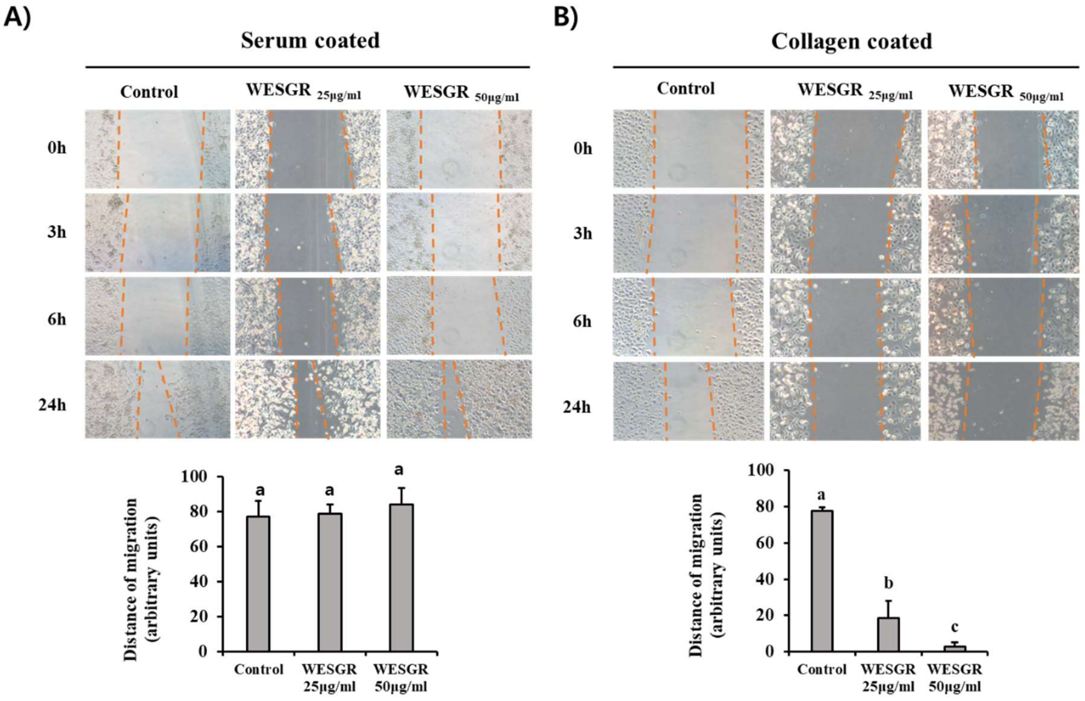

**Supplementary figure 2.** PC3 cells were seeded on the serum (A) or collagen (B) coated plate and then treated with and without WESGR. After treatement, PC3 cells were subjected to the scratch wound assay. The distance of the migration was measured using Image J program at 24h after a cratch wound was introduced. Migration of PC3 cells on serum coated plate did not affected by WESGR treatment. However, WESGR treatment attenuated the migration of PC3 cells on collagen coated plate. The upper panel is representative images and the lower panel shows the quntifiaction graph of the realative migration distance. All values are expressed as the mean ± standard deviation (SD) of triplicate measurements. Different letters indicate significant differences ($p < 0.05$).

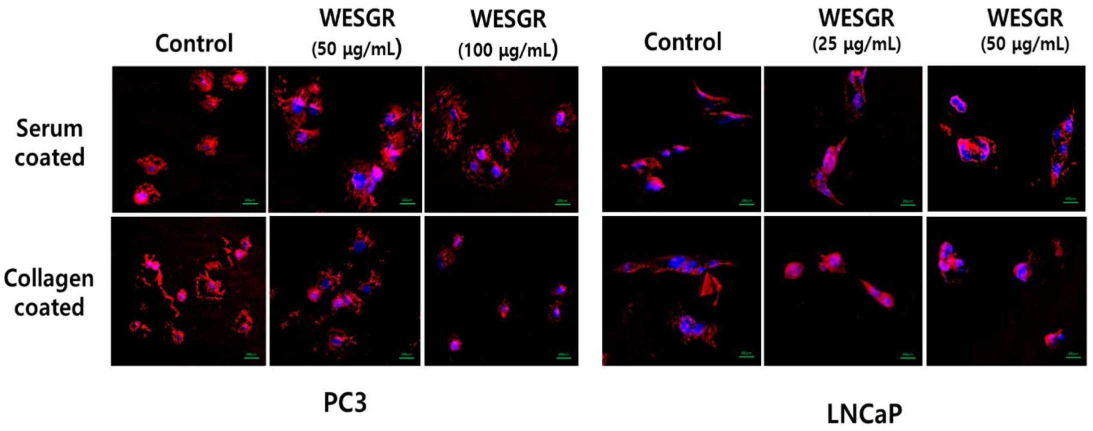


**Supplementary figure 3.** Treatment of WESGR attenuated collagen induced actin formation of PC3 and LNCaP cells. PC3 and LNCaP cells were pretreated with and without WESGR for 6 h and then seeded on serum or collagen coated cover glass. After further incubation for 30 min (PC3) or 3 h (LNCaP) at 37 °C in a $CO_2$ incubator, cells were fixed with 4% paraformaldehyde and then stained with Hoechst 33342 (blue) (Invitrogen, Carlsbad, CA, USA) and Tetramethylrhodamine-5-isothiocyanate (TRITC)-conjugated phalloidin (red) (Merck Millipore , Middlesex County, MA, USA) for 2 h at room temperature. Cells were washed twice with PBS, fluorescence images were taken with a confocal laser scanning microscope (Nikon, Tokyo, Japan).

© 2020 by the authors. Submitted for possible open access publication under the terms and conditions of the Creative Commons Attribution (CC BY) license (http://creativecommons.org/licenses/by/4.0/).
